# 4-(Pyridin-2-yl)-1,3-dithiol-2-one

**DOI:** 10.1107/S1600536812001407

**Published:** 2012-01-18

**Authors:** Guoquan Zhou, Xinzhi Chen

**Affiliations:** a Department of Chemical and Biological Engineering, Zhejiang University, Hangzhou 310027, People’s Republic of China; b College of Chemical Engineering, Ningbo University of Technology, Ningbo 315016, People’s Republic of China

## Abstract

In the title compound, C_8_H_5_NOS_2_, the non-H atoms are approximately coplanar [maxium deviation = 0.060 (3) Å]. The dihedral angle between the least-squares planes of the pyridine and 1,3-dithiol-2-one rings is 5.96 (17)°. The crystal packing is stabilized by weak inter­molecular C—H⋯O hydrogen bonds and by an S⋯S close contact [3.510 (5) Å].

## Related literature

For background to the chemistry of pyridine-based tetra­thia­fulvalenes, see: Fabre (2004 ▶); Zhu *et al.* (2010 ▶). For the preparation and crystal structures of related compounds, see: Zhu *et al.* (2010 ▶); Han *et al.* (2007 ▶).
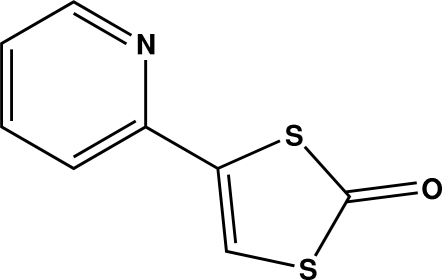


## Experimental

### 

#### Crystal data

C_8_H_5_NOS_2_
*M* *_r_* = 195.27
Orthorhombic, 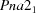

*a* = 11.157 (2) Å
*b* = 5.3216 (10) Å
*c* = 13.689 (3) Å
*V* = 812.8 (3) Å^3^
*Z* = 4
Mo *K*α radiation
μ = 0.60 mm^−1^
*T* = 223 K
0.60 × 0.25 × 0.20 mm

#### Data collection

Rigaku Saturn CCD diffractometer
Absorption correction: multi-scan (REQAB; Jacobson, 1998 ▶) *T*_min_ = 0.564, *T*_max_ = 0.887
2825 measured reflections
1215 independent reflections
1144 reflections with *I* > 2σ(*I*)
*R*_int_ = 0.027

#### Refinement

*R*[*F* ^2^ > 2σ(*F* ^2^)] = 0.032
*wR*(*F* ^2^) = 0.081
*S* = 1.10
1215 reflections
110 parameters
1 restraint
H-atom parameters constrained
Δρ_max_ = 0.18 e Å^−3^
Δρ_min_ = −0.23 e Å^−3^
Absolute structure: Flack (1983 ▶), 430 Friedel pairs
Flack parameter: −0.09 (11)

### 

Data collection: *CrystalClear* (Rigaku, 2005 ▶); cell refinement: *CrystalClear*; data reduction: *CrystalStructure* (Rigaku, 2005 ▶); program(s) used to solve structure: *SHELXS97* (Sheldrick, 2008 ▶); program(s) used to refine structure: *SHELXL97* (Sheldrick, 2008 ▶); molecular graphics: *ORTEP-3 for Windows* (Farrugia, 1997 ▶); software used to prepare material for publication: *SHELXTL* (Sheldrick, 2008 ▶).

## Supplementary Material

Crystal structure: contains datablock(s) I, global. DOI: 10.1107/S1600536812001407/pk2379sup1.cif

Structure factors: contains datablock(s) I. DOI: 10.1107/S1600536812001407/pk2379Isup2.hkl

Supplementary material file. DOI: 10.1107/S1600536812001407/pk2379Isup3.cml

Additional supplementary materials:  crystallographic information; 3D view; checkCIF report

## Figures and Tables

**Table 1:** Hydrogen-bond geometry (Å, °)

*D*—H⋯*A*	*D*—H	H⋯*A*	*D*⋯*A*	*D*—H⋯*A*
C7—H7⋯O1^i^	0.94	2.46	3.3486	158

Symmetry code: (i) 
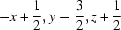
.

**Fig. 1.**
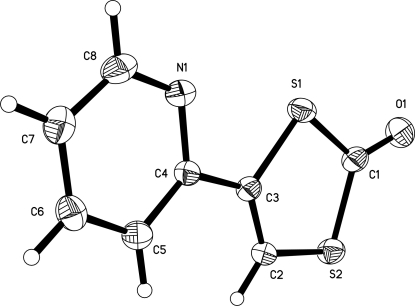
The molecular structure of the title compound. Displacement ellipoids are drawn at the 30% probability level.

**Fig. 2.**
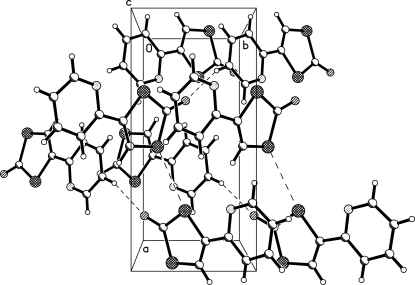
A crystal packing diagram viewed down the c axis. Dashed lines indicate the weak C—H···O interactions.

## supplementary crystallographic information

### Comment

Bifunctional molecules featuring a TTF (tetrathiafulvalene) unit with a pyridine, TTF-py, have been explored and a series of new TTF compounds with transition metal centers have been synthesized. The title compound is an intermediate for synthesis of this type of TTF derivative and also a donor-acceptor ligand.

In the title compound (Fig. 1), all bonds lengths and angles are found to be within the range for 4-pyridine-4-yl-1,3-dithiol-2-one (Han *et al.*, 2007). In addition, the non-H atoms are approximately planar [maxium deviation = 0.060 (3) Å] (Fig. 1). There are short S···S contacts [3.510 (5) Å] and weak C—H···O intermolecular hydrogen bonds in the crystal structure (Table 1, Fig.2).

### Experimental

The title compound was synthesized according to a literature procedure (Han *et al.*, 2007). Slow evaporation of a solution in THF gave single crystals suitable for *X*-ray analysis.

### Refinement

All H atoms were placed geometrically (C—H = 0.94 Å) with *U*_iso_ = 1.2*U*_eq_ of the parent atom.

### Crystal data

**Table:**

C_8_H_5_NOS_2_	*F*(000) = 400
*M**_r_* = 195.27	*D*_x_ = 1.596 Mg m^−^^3^
Orthorhombic, *P**n**a*2_1_	Mo *K*α radiation, λ = 0.71075 Å
Hall symbol: P 2c -2n	Cell parameters from 2396 reflections
*a* = 11.157 (2) Å	θ = 3.5–27.5°
*b* = 5.3216 (10) Å	µ = 0.60 mm^−^^1^
*c* = 13.689 (3) Å	*T* = 223 K
*V* = 812.8 (3) Å^3^	Block, colorless
*Z* = 4	0.60 × 0.25 × 0.20 mm

### Data collection

**Table:**

Rigaku Saturn CCD diffractometer	1215 independent reflections
Radiation source: fine-focus sealed tube	1144 reflections with *I* > 2σ(*I*)
graphite	*R*_int_ = 0.027
Detector resolution: 14.63 pixels mm^-1^	θ_max_ = 25.5°, θ_min_ = 3.9°
ω scans	*h* = −10→13
Absorption correction: multi-scan (REQAB; Jacobson, 1998)	*k* = −6→5
*T*_min_ = 0.564, *T*_max_ = 0.887	*l* = −15→16
2825 measured reflections	

### Refinement

**Table:**

Refinement on *F*^2^	Secondary atom site location: difference Fourier map
Least-squares matrix: full	Hydrogen site location: inferred from neighbouring sites
*R*[*F*^2^ > 2σ(*F*^2^)] = 0.032	H-atom parameters constrained
*wR*(*F*^2^) = 0.081	*w* = 1/[σ^2^(*F*_o_^2^) + (0.0458*P*)^2^ + 0.0545*P*] where *P* = (*F*_o_^2^ + 2*F*_c_^2^)/3
*S* = 1.10	(Δ/σ)_max_ = 0.001
1215 reflections	Δρ_max_ = 0.18 e Å^−^^3^
110 parameters	Δρ_min_ = −0.23 e Å^−^^3^
1 restraint	Absolute structure: Flack (1983), 430 Friedel pairs
Primary atom site location: structure-invariant direct methods	Flack parameter: −0.09 (11)

### Special details

**Table:**

Geometry. All e.s.d.'s (except the e.s.d. in the dihedral angle between two l.s. planes) are estimated using the full covariance matrix. The cell e.s.d.'s are taken into account individually in the estimation of e.s.d.'s in distances, angles and torsion angles; correlations between e.s.d.'s in cell parameters are only used when they are defined by crystal symmetry. An approximate (isotropic) treatment of cell e.s.d.'s is used for estimating e.s.d.'s involving l.s. planes.
Refinement. Refinement of *F*^2^ against ALL reflections. The weighted *R*-factor *wR* and goodness of fit *S* are based on *F*^2^, conventional *R*-factors *R* are based on *F*, with *F* set to zero for negative *F*^2^. The threshold expression of *F*^2^ > σ(*F*^2^) is used only for calculating *R*-factors(gt) *etc*. and is not relevant to the choice of reflections for refinement. *R*-factors based on *F*^2^ are statistically about twice as large as those based on *F*, and *R*- factors based on ALL data will be even larger.

### Fractional atomic coordinates and isotropic or equivalent isotropic displacement parameters (Å^2^)

**Table:**

	*x*	*y*	*z*	*U*_iso_*/*U*_eq_	
S1	0.29937 (7)	1.06740 (15)	0.65200 (6)	0.0415 (2)	
S2	0.53107 (7)	1.18329 (17)	0.55954 (7)	0.0439 (2)	
O1	0.3307 (3)	1.4374 (5)	0.52711 (19)	0.0562 (7)	
N1	0.2818 (2)	0.6783 (5)	0.7860 (2)	0.0407 (7)	
C3	0.4232 (3)	0.8896 (6)	0.6876 (2)	0.0329 (7)	
C4	0.3981 (3)	0.6970 (6)	0.7623 (2)	0.0328 (7)	
C8	0.2525 (4)	0.5068 (7)	0.8529 (3)	0.0482 (9)	
H8	0.1713	0.4931	0.8704	0.058*	
C7	0.3323 (4)	0.3487 (7)	0.8980 (3)	0.0474 (9)	
H7	0.3068	0.2274	0.9434	0.057*	
C6	0.4513 (4)	0.3754 (7)	0.8740 (3)	0.0443 (8)	
H6	0.5091	0.2738	0.9047	0.053*	
C5	0.4867 (3)	0.5504 (6)	0.8052 (3)	0.0380 (8)	
H5	0.5678	0.5695	0.7880	0.046*	
C2	0.5273 (3)	0.9427 (6)	0.6451 (2)	0.0349 (7)	
H2	0.5970	0.8523	0.6609	0.042*	
C1	0.3772 (3)	1.2650 (6)	0.5707 (2)	0.0414 (8)	

### Atomic displacement parameters (Å^2^)

**Table:**

	*U*^11^	*U*^22^	*U*^33^	*U*^12^	*U*^13^	*U*^23^
S1	0.0314 (4)	0.0446 (5)	0.0484 (5)	0.0045 (3)	−0.0023 (4)	0.0054 (5)
S2	0.0388 (5)	0.0469 (5)	0.0459 (4)	−0.0031 (4)	−0.0006 (4)	0.0105 (4)
O1	0.0569 (16)	0.0481 (15)	0.0636 (18)	0.0060 (12)	−0.0125 (14)	0.0169 (13)
N1	0.0335 (15)	0.0428 (17)	0.0456 (16)	−0.0022 (13)	0.0034 (13)	0.0024 (15)
C3	0.0306 (17)	0.0326 (16)	0.0354 (16)	−0.0010 (14)	−0.0022 (13)	−0.0021 (14)
C4	0.0295 (16)	0.0326 (17)	0.0362 (16)	0.0008 (14)	−0.0022 (14)	−0.0008 (14)
C8	0.0369 (18)	0.057 (2)	0.051 (2)	−0.0075 (19)	0.0089 (17)	−0.001 (2)
C7	0.057 (2)	0.0413 (19)	0.044 (2)	−0.0030 (19)	0.0045 (17)	0.0009 (17)
C6	0.051 (2)	0.0404 (18)	0.0418 (18)	0.0073 (17)	−0.0046 (16)	0.0032 (17)
C5	0.0345 (18)	0.041 (2)	0.0383 (18)	−0.0006 (17)	0.0015 (14)	−0.0001 (16)
C2	0.0292 (15)	0.0358 (16)	0.0397 (18)	0.0020 (13)	−0.0029 (17)	0.0013 (15)
C1	0.0367 (17)	0.0442 (18)	0.0433 (18)	−0.0036 (15)	−0.0062 (17)	−0.0061 (18)

### Geometric parameters (Å, °)

**Table:**

S1—C3	1.744 (3)	C4—C5	1.390 (5)
S1—C1	1.760 (4)	C8—C7	1.372 (5)
S2—C2	1.736 (3)	C8—H8	0.9400
S2—C1	1.778 (4)	C7—C6	1.375 (6)
O1—C1	1.211 (4)	C7—H7	0.9400
N1—C8	1.334 (5)	C6—C5	1.382 (5)
N1—C4	1.342 (4)	C6—H6	0.9400
C3—C2	1.329 (5)	C5—H5	0.9400
C3—C4	1.474 (4)	C2—H2	0.9400
			
C3—S1—C1	96.32 (17)	C6—C7—H7	121.4
C2—S2—C1	95.66 (16)	C7—C6—C5	120.5 (4)
C8—N1—C4	117.0 (3)	C7—C6—H6	119.7
C2—C3—C4	128.1 (3)	C5—C6—H6	119.7
C2—C3—S1	117.0 (2)	C6—C5—C4	117.6 (3)
C4—C3—S1	114.8 (2)	C6—C5—H5	121.2
N1—C4—C5	123.0 (3)	C4—C5—H5	121.2
N1—C4—C3	113.8 (3)	C3—C2—S2	118.3 (2)
C5—C4—C3	123.2 (3)	C3—C2—H2	120.9
N1—C8—C7	124.7 (4)	S2—C2—H2	120.9
N1—C8—H8	117.6	O1—C1—S1	123.6 (3)
C7—C8—H8	117.6	O1—C1—S2	123.8 (3)
C8—C7—C6	117.1 (4)	S1—C1—S2	112.63 (19)
C8—C7—H7	121.4		
			
C1—S1—C3—C2	−2.3 (3)	C7—C6—C5—C4	0.3 (6)
C1—S1—C3—C4	177.7 (2)	N1—C4—C5—C6	1.2 (5)
C8—N1—C4—C5	−1.1 (5)	C3—C4—C5—C6	−179.7 (3)
C8—N1—C4—C3	179.7 (3)	C4—C3—C2—S2	−179.5 (2)
C2—C3—C4—N1	−175.8 (3)	S1—C3—C2—S2	0.5 (4)
S1—C3—C4—N1	4.3 (4)	C1—S2—C2—C3	1.5 (3)
C2—C3—C4—C5	5.1 (5)	C3—S1—C1—O1	−177.0 (3)
S1—C3—C4—C5	−174.9 (3)	C3—S1—C1—S2	3.1 (2)
C4—N1—C8—C7	−0.4 (5)	C2—S2—C1—O1	177.2 (3)
N1—C8—C7—C6	1.9 (6)	C2—S2—C1—S1	−2.9 (2)
C8—C7—C6—C5	−1.7 (6)		

### Hydrogen-bond geometry (Å, °)

**Table:**

*D*—H···*A*	*D*—H	H···*A*	*D*···*A*	*D*—H···*A*
C7—H7···O1^i^	0.94	2.46	3.3486	158

Symmetry codes: (i) −*x*+1/2, *y*−3/2, *z*+1/2.
